# Availability and use of PET in patients with brain tumours – a European Organisation for Research and Treatment of Cancer - Brain Tumour Group (EORTC-BTG) survey

**DOI:** 10.1007/s00259-025-07366-0

**Published:** 2025-06-04

**Authors:** Maximilian J. Mair, Philipp Lohmann, Norbert Galldiks, Mattias Belting, Petter Brandal, Martinus P. G. Broen, Francesco Cicone, Jean-François Daisne, François Ducray, Felix Ehret, Julia Furtner, Asgeir S. Jakola, Maximilian Niyazi, Alessia Pellerino, Marika Rasschaert, Evangelia Razis, Felix Sahm, Marion Smits, Nelleke Tolboom, Antoine Verger, Emilie Le Rhun, Giuseppe Minniti, Michael Weller, Matthias Preusser, Nathalie L. Albert

**Affiliations:** 1 Department of Nuclear Medicine, LMU University Hospital, Ludwig Maximilians University, Marchioninistraße 15, 81377 Munich, Germany; 2 Division of Oncology, Department of Medicine I, Medical University of Vienna, Vienna, Austria; 3 Institute of Neuroscience and Medicine (INM-3, INM-4), Research Center Juelich, Juelich, Germany; 4 Department of Nuclear Medicine, RWTH Aachen University Hospital, Aachen, Germany; 5 Department of Neurology, Faculty of Medicine and University Hospital Cologne, Cologne, Germany; 6 Division of Oncology, Department of Clinical Sciences, Lund University, Lund, Sweden; 7 Department of Hematology, Oncology and Radiophysics, Skåne University Hospital, Lund, Sweden; 8 Department of Oncology, Oslo University Hospital, Oslo, Norway; 9 Institute for Clinical Medicine, University of Oslo, Oslo, Norway; 10 Department of Neurology, GROW School for Oncology and Reproduction, Maastricht University Medical Centre, Maastricht, The Netherlands; 11 Nuclear Medicine Unit, Department of Experimental and Clinical Medicine, “Magna Graecia” University of Catanzaro, Catanzaro, Italy; 12 Department of Radiation Oncology, Iridium Cancer Network, Antwerp, Wilrijk Belgium; 13 Department of Neurooncology, Hôpital Neurologique Pierre Wertheimer, Hospices Civils de Lyon, Bron, France; 14 Université Claude Bernard Lyon 1, Villeurbanne, France; 15 Department of Radiation Oncology, Charité – Universitätsmedizin Berlin, Corporate Member of Freie Universität Berlin and Humboldt-Universität Zu Berlin, Berlin, Germany; 16 German Cancer Consortium (DKTK), Partner Site Berlin, a partnership between DKFZ and Charité – Universitätsmedizin Berlin, Berlin, Germany; 17 Research Center for Medical Image Analysis and Artificial Intelligence (MIAAI), Faculty of Medicine and Dentistry, Danube Private University, Krems, Austria; 18 Department of Neurosurgery, Sahlgrenska University Hospital, Gothenburg, Sweden; 19 Institute of Neuroscience and Physiology, Department of Clinical Neuroscience, Sahlgrenska Academy, Gothenburg, Sweden; 20 Department of Radiation Oncology, University Hospital Tuebingen, Tuebingen, Germany; 21 Division of Neuro-Oncology, Department of Neuroscience “Rita Levi Montalcini”, University and City of Health and Science Hospital, Torino, Italy; 22 Department of Medical Oncology, University Hospital Antwerp, Edegem, Belgium; 23 Third Department of Medical Oncology, Hygeia Hospital, Athens, Greece; 24 Department of Neuropathology, Institute of Pathology, Clinical Cooperation Unit Neuropathology, German Cancer Research Center (DKFZ), Ruprecht-Karls University Heidelberg, and German Consortium for Translational Cancer Research (DKTK), Heidelberg, Germany; 25 Department of Radiology & Nuclear Medicine, Erasmus MC, University Medical Centre Rotterdam, Rotterdam, The Netherlands; 26 Brain Tumour Centre, Erasmus MC Cancer Institute, Rotterdam, The Netherlands; 27 Department of Radiology and Nuclear Medicine, University Medical Centre Utrecht, Utrecht, The Netherlands; 28 Department of Nuclear Medicine & Nancyclotep Imaging Platform, CHRU Nancy and IADI INSERM, UMR 1254, Université de Lorraine, Nancy, France; 29 Department of Medical Oncology and Hematology, Brain Tumor Center, University Hospital and University of Zurich, Zurich, Switzerland; 30 Department of Radiological Sciences, Oncology and Anatomical Pathology, Sapienza University of Rome, Rome, Italy; 31 IRCCS Neuromed, Pozzilli, IS Italy; 32 Department of Neurology and Brain Tumor Center, University Hospital and University of Zurich, Zurich, Switzerland

**Keywords:** Positron emission tomography, Imaging, Glioma, Meningioma, Brain metastasis

## Abstract

**Purpose:**

Positron emission tomography (PET) is increasingly used in neuro-oncology. However, little is known about its application across European institutions and reasons for variable implementation.

**Methods:**

Between June and August 2024, members of the European Organisation for Research and Treatment of Cancer - Brain Tumour Group (EORTC-BTG) completed a cross-sectional online survey on PET use in neuro-oncological practice.

**Results:**

Overall, 103 replies from 20 countries were received. A PET facility was available at 96/103 (93.2%) sites, of whom 74 (77.1%) performed PET in patients with brain tumours. Reasons for not performing PET included limited availability of tracers (14/29, 48.3%), high cost (11/29, 37.9%), and PET perceived unnecessary (8/29, 27.6%). Of sites performing PET, 69/74 (93.2%) reported use in glioma, 58/74 (78.4%) in brain metastasis, 52/74 (70.3%) in meningioma, and 46/74 (62.2%) in CNS lymphoma. Amino acid PET was performed at 62/71 centres (87.3%; 3 not reported [n.r.]), most frequently in glioma (58/59, 98.3%, 3 n.r.) and for differentiation of treatment-related changes from tumour progression (58/59, 98.3%). Somatostatin receptor (SSTR) PET was performed at 50/68 sites (73.5%, 6 n.r.), mainly in meningioma (48/49, 98.0%), for patient selection before radioligand therapy (41/49, 83.7%) and for radiotherapy target volume definition (33/49, 67.3%). Unrestricted coverage by statutory health insurance was reported by 46/59 (78.0%) centres for amino acid PET and 33/49 (67.3%) for SSTR PET.

**Conclusion:**

PET use in neuro-oncology is variable across EORTC-BTG sites. Generation of evidence in clinical trials and surveys including non-academic institutions are needed to guide implementation in clinical practice.

**Supplementary Information:**

The online version contains supplementary material available at 10.1007/s00259-025-07366-0.

## Introduction

Central nervous system (CNS) tumours represent a heterogeneous group of neoplasms and are overall associated with variable impaired quality of life and frequently poor prognosis. Treatment usually consists of a multimodal approach including surgery, radiotherapy, and systemic treatment [1–3]. Throughout the clinical course, imaging plays a pivotal role in treatment planning and follow-up. Most frequently, structural magnetic resonance imaging (MRI) is performed given its superior soft tissue contrast compared to computed tomography (CT). However, treatment-related changes complicate the interpretability of morphological imaging even when advanced MRI techniques are used. Therefore, positron emission tomography (PET) is increasingly employed, as this improves the delineation of metabolically active tumour tissue independent of structural tissue changes. In extracranial disease, [^18^F]−2-fluoro-2-deoxy-D-glucose ([^18^F]FDG) PET is widely used as it allows detection of metabolically active areas [4]. However, its value in brain tumours is limited due to physiologically high glucose uptake of the brain and the resulting limited tumour-to-background contrast. Given the high expression of amino acid transporters in many brain tumours notably gliomas, amino acid tracers such as O-(2-[^18^F]-fluoroethyl)-L-tyrosine ([^18^F]FET), [^11^C]-methyl-L-methionine ([^11^C]MET), and 3,4-dihydroxy-6-[^18^F]-fluoro-L-phenylalanine ([^18^F]FDOPA) are preferably used [5, 6]. In meningioma, the abundant expression of somatostatin receptors (SSTR) is harnessed, and radiolabelled SSTR ligands such as [^68^Ga]gallium-DOTA-Tyr3-octreotate ([^68^Ga]Ga-DOTATATE), [^68^Ga]Ga-DOTA-Tyr3-octreotide ([^68^Ga]Ga-DOTATOC), and [^68^Ga]Ga-DOTA-1-NaI(3)-octreotide ([^68^Ga]Ga-DOTANOC) are applied [7].

The value of PET in neuro-oncology is underlined by the recent publication of consensus statements proposed by both nuclear medicine as well as neuro-oncological societies and expert panels [5–8]. Moreover, PET-based response assessment frameworks for gliomas (PET RANO 1.0) and brain metastases (PET RANO BM 1.0) have been developed, allowing standardized interpretation ready for implementation as endpoints in clinical trials [9, 10]. However, the presence of nuclear medicine facilities as well as approval, availability and reimbursement of tracers beyond [^18^F]FDG make use in clinical routine highly variable. To evaluate these factors, we performed a web-based survey among neuro-oncological centres of the European Organisation for Research and Treatment of Cancer – Brain Tumour Group (EORTC-BTG).

## Materials and methods

### Survey design

A web-based, structured, cross-sectional anonymous survey was developed by members of the EORTC-BTG Nuclear Medicine and Quality Assurance Committees. The survey was conducted using SurveyMonkey (www.surveymonkey.com, Dublin, Ireland), and the link for participation was sent via email to all EORTC members affiliated with centres of the EORTC-BTG network. Survey completion was allowed from June 12th to August 2nd, 2024, and further reminders were sent over the survey period. Participation or input by the local nuclear medicine physician was encouraged in the invitation letter, but no independent validation of replies by local nuclear medicine physicians was performed, and nuclear medicine physicians were only contacted directly if they were individual EORTC members affiliated with an EORTC-BTG site.

Questions were closed-ended with predefined response options. For questions with “other/not specified” options, the addition of further comments was allowed in a free-text field. The survey comprised centre-specific questions such as name and/or EORTC site number, medical specialty of the participant, number of managed patients with a brain tumour per year, availability of PET imaging and tracers, and presence of European Association of Nuclear Medicine (EANM) Research GmbH (EARL) accreditation (Supplementary Table S1). If a centre reported that PET was not used in patients with brain tumours, underlying reasons were inquired. For specific information on PET in patients with brain tumours, use of PET in distinct entities (glioma, brain metastasis, meningioma, and CNS lymphoma) was queried. For each tracer used in neuro-oncology ([^18^F]FDG, amino acid tracers, SSTR-targeted tracers), the number of scans per year, frequency according to entities and indications, availability and origin of tracers (commercial vs. locally manufactured), as well as statutory health insurance coverage were polled.

### Data processing and statistical analysis

Data were retrieved from SurveyMonkey using the built-in export feature. Replies from outside the EORTC geographical legal area (Europe and the Middle East), as well as those lacking unequivocal information on site location, were removed. Countries were categorized according to European subregions as defined by EuroVoc classification [11]. Duplicate responses from identical centres were consolidated, where preference was given to complete replies and those entered by nuclear medicine physicians. All categorical data are given as absolute numbers and percentages, whereas metric variables are given as numbers with ranges. Missing replies are reported accordingly.

Statistical analysis was performed using GraphPad Prism 10 (GraphPad Software, La Jolla, CA, USA) and R 4.4.1 (The R Foundation for Statistical Computing, Vienna, Austria) with RStudio 2024.09.1+394 (Posit PBC, Boston, MA, USA) and the packages *tidyverse*,* ggplot2*,* ggpubr*,* tidygeocoder*,* rnaturalearth*,* rnaturalearthdata*,* countrycode* and *ggrepel.*

## Results

### Baseline characteristics of participating centres

**Fig. 1:**
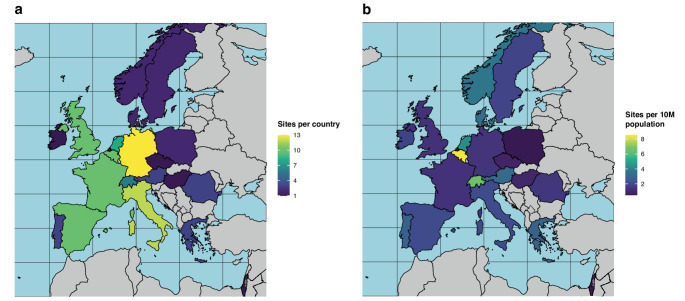
Map of numbers of participating sites per country in (**a**) absolute numbers and (**b**) per 10 million population. Countries without participating site are coloured in grey

The survey was distributed to 644 EORTC-BTG members from 312 sites, and 103 replies from 20 countries were recorded (Table 1; Fig. 1). Most were from Western European (61/103, 59.2%) and high-volume centres treating more than 100 patients with brain tumours per year (48/103, 46.6%). Most participants were nuclear medicine physicians (32/103, 31.1%), followed by radiation oncologists (26/103, 25.2%), medical oncologists (18/103, 17.5%), neurologists/neuro-oncologists (13/103, 12.5%), neurosurgeons (7/103, 6.8%), and others (7/103, 6.8%).

**Table 1:** Baseline characteristics of participating centres. ^1^ subregions according to European union EuroVoc geographical classification [11]

	Responses (*n* = 103)	Invited (*n* = 312)	Response rate per subregion/country [%]
Size of centre (managed patients with brain tumours per year)			
- ≤ 50	29 (28.2%)	-	
- 51–100	22 (21.4%)	-	
- > 100	48 (46.6%)	-	
- None	1 (1.0%)	-	
- *Not known*	*3 (2.9%)*	*-*	
**Subregions**^**1**^**and countries**			
- ***Western Europe***	***61 (59.2%)***	***177 (56.7%)***	***34.5%***
- Germany	13 (12.6%)	43 (13.8%)	30.2%
- Belgium	10 (9.7%)	24 (7.7%)	41.7%
- France	10 (9.7%)	35 (11.2%)	28.6%
- United Kingdom	10 (9.7%)	31 (9.9%)	32.3%
- The Netherlands	8 (7.8%)	17 (5.4%)	47.1%
- Switzerland	6 (4.9%)	16 (5.1%)	37.5%
- Austria	3 (2.9%)	7 (2.2%)	42.9%
- Ireland	1 (1.0%)	4 (1.3%)	25.0%
- ***Southern Europe***	***28 (27.2%)***	***80 (25.6%)***	***35.0%***
- Italy	12 (11.7%)	36 (11.5%)	33.3%
- Spain	10 (9.7%)	31 (9.9%)	32.3%
- Greece	3 (2.9%)	5 (1.6%)	60.0%
- Portugal	3 (2.9%)	8 (2.6%)	37.5%
- ***Central and Eastern Europe***	***7 (6.8%)***	***14 (4.5%)***	***50.0%***
- Romania	3 (2.9%)	4 (1.3%)	75.0%
- Poland	2 (1.9%)	6 (1.9%)	33.3%
- Czech Republic	1 (1.0%)	1 (0.3%)	100.0%
- Hungary	1 (1.0%)	3 (0.9%)	33.3%
- ***Northern Europe***	***6 (4.9%)***	***12 (3.8%)***	***50.0%***
- Denmark	2 (1.9%)	4 (1.3%)	50.0%
- Norway	2 (1.9%)	4 (1.3%)	50.0%
- Sweden	2 (1.9%)	4 (1.3%)	50.0%
- ***Middle East***	***1 (1.0%)***	***4 (1.3%)***	***25.0%***
- Israel	1 (1.0%)	4 (1.3%)	25.0%
- *Others (outside EORTC geographical legal area or countries with no participating site)*		25 (8.0%)	-
**Medical specialty of respondent**			
- Nuclear medicine	32 (31.1%)	-	
- Radiation Oncology	26 (25.2%)	-	
- Medical Oncology	18 (17.5%)	-	
- Neurology/Neuro-Oncology	13 (12.6%)	-	
- Neurosurgery	7 (6.8%)	-	
- Clinical Oncology	3 (2.9%)	-	
- (Neuro-)Radiology	2 (1.9%)	-	
- *Other*	*2 (1.9%)*	*-*

### Availability of PET scanners and use in brain tumour entities

**Fig. 2:**
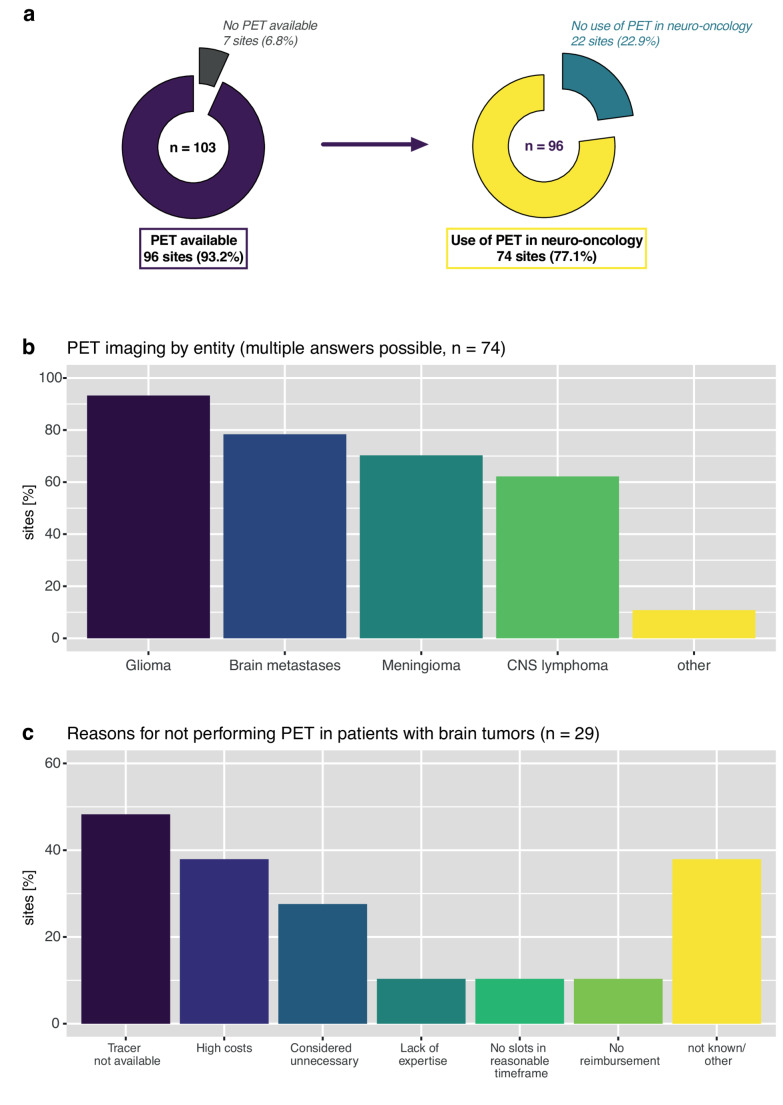
Use of PET at participating brain tumour centres. (**a**) Availability of PET and use of PET in patients with brain tumours; (**b**) Fractions of sites performing PET imaging by brain tumour entity; (**c**) Reasons for not performing PET in patients with brain tumours. Multiple answers possible in (**b**) and (**c**). Abbreviations: CNS = Central Nervous System; PET = positron emission tomography

At least one PET modality (either PET-CT, PET-MRI, or PET only) was available at 96/103 (93.2%) sites. PET-CT was present at 91/103 (88.3%) sites, whereas PET-MRI scanners were available at 27/103 (26.2%) sites, and both PET-CT/PET-MRI at 26/103 (25.2%) sites. A lack of any PET facility was reported by 7/103 (6.8%) sites (Fig. 2A), of which the majority (5/7, 71.4%) managed 50 or fewer patients with brain tumours per year. Excluding 43/103 (42.7%) sites with unknown EARL accreditation status or missing information, 44/60 (73.3%) sites declared being EARL-accredited for fluorine-18 (^18^F), 31/60 (45.6%) for gallium-68 (^68^Ga), and 30/60 (50.0%) for both, whereas none was reported by 15/60 (25.0%).

Of 96 sites with available PET scanner, 74 (77.1%) performed PET in patients with brain tumours. Of these, PET was performed in patients with glioma in 69/74 (93.2%) centres, patients with brain metastasis at 58/74 (78.4%), patients with meningioma at 52/74 (70.3%), and patients with CNS lymphoma at 46/74 (62.2%) sites (Fig. 2B). Overall, 33/74 (44.6%) sites performed PET in all these entities. Other (8 free text entries provided) included ependymomas, pituitary adenomas, pilocytic astrocytomas, and other rare tumours (unspecified).

Of 29 centres not performing PET in patients with brain tumours, provided reasons were limited availability or implementation of tracers (14/29, 48.3%), high cost (11/29, 37.9%), PET considered unnecessary (8/29, 27.6%), and limited capacity as well as lack of expertise in PET interpretation, lack of reimbursement by health insurance, or unknown in 3/29 (10.3%), each (Fig. 2C). Further free-text entries included reliance on advanced MRI (*n* = 2), PET facilities not currently available but planned in the future, effective contracts with external institutions, or governmental restrictions on the number of PET facilities (*n* = 1, each). Only two responses to this question were provided by nuclear medicine physicians, who reported limited availability of tracers as main reason.

### Use of amino acid PET in patients with brain tumours

**Fig. 3:**
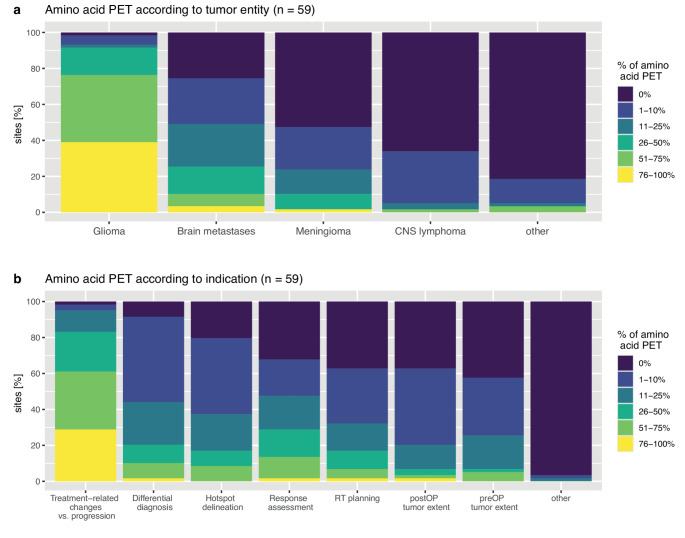
Percentages of amino acid PET examinations per (**a**) entity and (**b**) indication at sites as estimated by survey participants. Abbreviations: CNS = Central Nervous System; PET = positron emission tomography; preOP/postOP = pre-/postoperative; RT = radiotherapy

Of 71 completed replies in this Sect. (3 missing), amino acid PET was performed at 62 (87.3%) centres, with a median number of 50 (range: 3–900) scans per year. Out of these, 59 respondents provided further information on entities and indications (Fig. 3A/B). Amino acid PET was performed by 58/59 (98.3%) centres in patients with glioma, followed by 44/59 (74.6%) in patients with brain metastasis, 28/59 (47.5%) in patients with meningioma, 20/59 (33.9%) in patients with CNS lymphoma, and 11/59 (18.6%) in patients with other entities. These included indeterminate/rare entities (not specified; *n* = 3), pituitary adenoma (*n* = 2), as well as ependymoma and pilocytic astrocytoma (*n* = 1, each). Overall, the most prevalent indication was the differentiation of treatment-related changes from tumour progression (58/59, 98.3%), followed by differential diagnosis (54/59, 91.5%), hotspot delineation (47/59, 79.7%), response assessment (40/59, 67.8%), as well as evaluation of postoperative tumour volume and radiotherapy target volume definition (each 37/59, 62.7%). The use of standardized response assessment criteria such as PET RANO 1.0 was reported by 19/59 (32.2%) sites in clinical routine and 13/59 (22.0%) in clinical trial settings.

With 45/59 (76.3%) centres, [^18^F]FET was the most widely used tracer, followed by [^18^F]FDOPA at 22/59 (37.3%) and [^11^C]MET at 15/59 (25.4%) sites (more than one answer allowed; Supplementary Figure S1). At two centres (3.4%), [^11^C]choline was applied. Two or more of these tracers were used at 22/59 (37.3%) sites. A commercially available tracer was employed at 28/59 (47.5%) centers, whereas a locally manufactured compound was used at 21/59 (35.6%), and both at 5/59 (8.5%), while tracer provenience was unknown at 5/59 (8.5%) sites. Overall, 35/59 (59.3%) reported use of dynamic acquisition protocols. Coverage by statutory health insurance was reported by 46/59 (78.0%) centres (unknown at 5/59, 6.8%), with 4/54 (7.4%) each stating conditional coverage or a lack of reimbursement. The latter were located in Germany, the Netherlands, Poland, and Switzerland; however, other sites in these countries reported coverage, suggesting local rather than country-specific policies.

### Use of SSTR PET in patients with brain tumours

**Fig. 4:**
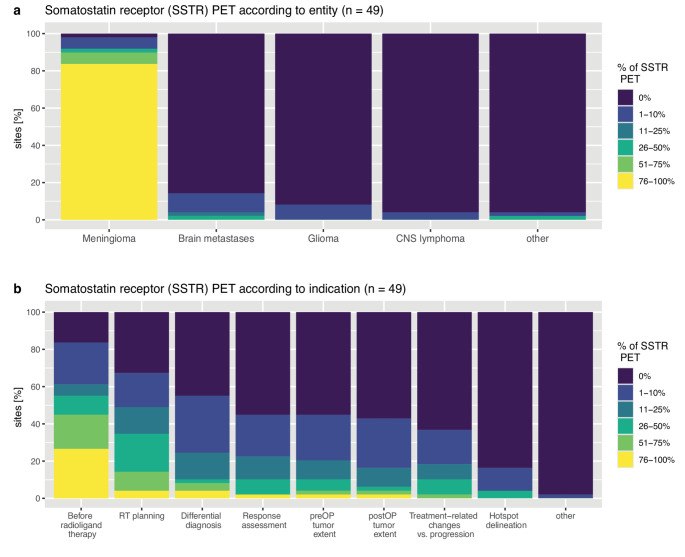
Percentages of SSTR PET examinations per (**a**) entity and (**b**) indication at sites as estimated by survey participants. Abbreviations: CNS = Central Nervous System; PET = positron emission tomography; preOP/postOP = pre-/postoperative; RT = radiotherapy

Of 68 completed replies in this Sect. (6 missing), SSTR PET was performed at 50 (73.5%) sites, with a median number of 14 (range: 1–150) scans per year. Of these, 49 (98.0%) sites provided further information on entities and indications. SSTR PET was predominantly performed in meningioma (48/49, 98.0%) and only rarely in other entities (Fig. 4A). SSTR PET was mainly done for patient selection for radioligand therapy (41/49, 83.7%), followed by radiotherapy target volume definition (33/49, 67.3%) and differential diagnosis (27/49, 55.1%; Fig. 4B). Of note, in the 6 centres performing the highest number of SSTR PETs (≥ 50 per year), radiotherapy target delineation was the most common indication.

Used tracers (multiple answers possible) were [^68^Ga]Ga-DOTATOC at 28/49 (57.1%) institutions, followed by [^68^Ga]Ga-DOTATATE at 16/49 (32.7%), [^68^Ga]Ga-DOTANOC at 3/49 (6.1%), and SiFAlin-tagged [Tyr^3^]-octreotate ([^18^F]F-SiTATE) as well as [^18^F]AlF-1,4,7-triazacyclononane-1,4,7-triacetate ([^18^F]AlF-NOTA)-octreotide or unknown at 2/49 (4.1%) sites, each. Locally manufactured tracers were used at 25/49 (51.0%) centres, whereas a commercial tracer was applied at 14/49 (28.6%) and both at 5/49 (10.2%), while tracer origin was unknown at 5/49 (10.2%) centres. In general, only head scans were performed (25/49, 51.0%), whereas in 10/49 (20.4%) centers, whole-body acquisition was performed on a regular basis (unknown in 3/49, 6.1%). Furthermore, 11/49 (22.4%) performed whole-body scans in individual cases, such as for assessment of extracranial disease (either in meningioma or neuroendocrine tumour brain metastasis) at 7/11 (63.3%) sites, planning of radioligand therapy (including evaluation of tumour-to-liver ratio) at 3/11 (27.3%), and logistic reasons at 1/11 (9.1%). Costs of SSTR PET were reimbursed at 33/49 (67.3%) sites (6/49, 12.2% unknown), whereas lack of or conditional coverage was reported by 5/49 (10.2%) centres, each. The latter were located in Austria, Switzerland, the Czech Republic, Germany, the Netherlands and the United Kingdom. As with amino acid PET, other sites in most of these countries reported coverage, again indicating local rather than country-specific policies.

### Use of [^18^F]FDG PET in patients with brain tumours

[^18^F]FDG PET was performed at 26/74 (35.1%) centres in patients with brain tumours, and the median number of scans per year was 20 (range: 3–5100). Only 1/68 (1.5%) reported use of [^18^F]FDG PET with lack of amino acid and SSTR PET, while all tracers were used at 21/68 (30.9%) sites (6 n.r.). The distribution of entities and frequent indications is illustrated in Supplementary Figure S2A/B (3/26 [11.5%] n.r.). Most centres indicated use of [^18^F]FDG PET in patients with CNS lymphoma (22/23, 95.7%) followed by brain metastasis (20/23, 87.0%). [^18^F]FDG PET was used for differential diagnosis at 20/23 (87.0%) sites, followed by response assessment (14/23, 60.9%), hotspot delineation (14/23, 60.9%), and the differentiation of treatment-related changes from tumour progression (13/23, 56.5%). Further free-text entries included evaluation of extracranial disease in patients with lymphoma and brain metastasis (*n* = 4).

Commercial [^18^F]FDG PET tracers were used at 14/23 (60.9%) centres, whereas 5/23 (21.7%) sites applied locally manufactured tracers, and 4/23 (17.4%) used both. Reimbursement of [^18^F]FDG PET examinations by statutory health insurance was reported by 22/23 (95.7%) sites (1 unknown).

## Discussion

While PET is increasingly used in neuro-oncology, multi-institutional data on its application in distinct entities, employed tracers, and clinical indications are scarce. The present data provide an overview on PET use at more than 100 European neuro-oncology centres of the EORTC-BTG network. The results of the present survey indicate highly variable patterns of use across brain tumour entities, indications and sites, suggesting differing practice between institutions, but also underlying operational and economic differences across diverse healthcare frameworks in Europe.

The use of PET in patients with brain tumours was reported by 77% of sites equipped with a nuclear medicine facility. Among those, amino acid PET was most widely used, particularly in glioma, followed by SSTR PET in meningioma. In contrast, only 35% of participating sites reported the use of [^18^F]FDG PET in neuro-oncology, mainly for the evaluation of extracranial disease in patients with brain metastasis and lymphoma. As the improved tumour-to-brain ratio of radiolabelled amino acids is well established, the use of [^18^F]FDG is generally discouraged for primary brain tumour imaging by current guidelines and procedure standards [5, 6, 9]. Under certain circumstances and particularly in the US, [^18^F]FDG PET is still performed due to the limited access to amino acid PET tracers for brain tumour imaging [12]. An application for FDA approval of a commercial [^18^F]FET compound (TLX101-CDx) is currently under review [13]. Thereby, an increased use of [^18^F]FET is also expected in the US, where currently also [^18^F]FDOPA and [^18^F]fluciclovine are applied as they are approved in other indications or have orphan drug designation for glioma imaging. In contrast, participating sites in the present European survey mainly reported the use of [^18^F]FET, [^18^F]FDOPA and [^11^C]MET, which are also the most widely used amino acid tracers in the literature [9].

Indeed, the limited availability of tracers was among the most frequently reported reasons for not implementing PET in this survey. Other reasons were multifactorial and included high cost as well as PET not perceived necessary. While institutional factors may contribute, many of these reported reasons can be attributed to insufficient evidence. Most available data are based on small, mostly single-centre and retrospective correlative studies demonstrating an association of PET uptake with active tumour tissue and malignancy [14–17]. While PET has proven helpful in differential diagnosis, planning of local therapies, and response assessment in various entities, there remains a lack of prospective trials assessing whether the incorporation of PET in decision-making translates to an added value in the clinical management, ideally with improved outcomes. Particularly in glioma, promising signals were observed, although mostly in uncontrolled or non-randomized clinical trials. For instance, [^18^F]FDOPA PET-assisted re-irradiation in progressive glioblastoma resulted in a three-month progression-free survival (PFS) of 85% in a small, uncontrolled phase 2 trial [18]. Along these lines, patients with [^18^F]FDOPA PET-assisted dose-escalated radiotherapy for newly diagnosed glioblastoma compared favourably to institutional controls in terms of PFS and overall survival (OS) in a phase 2 trial [19]. However, among the few randomized controlled trials, the GLIAA/NOA-10 trial failed to show a survival benefit for PET-based reirradiation in progressive glioblastoma compared to MRI-based radiotherapy planning [20]. In this study, all patients received [^18^F]FET PET at baseline excluding the treatment of radiation necrosis. However, the clinical benefit of reirradiation in progressive glioblastoma remains controversial [21, 22] and is currently being re-evaluated in the EORTC-2227 (LEGATO) trial, which is though based on MRI only [23]. The lack of robust evidence derived from well-designed controlled trials extends to other indications, such as PET-based surgery planning and response assessment in patients with glioma and other brain tumours. Overall, the generation of high-level evidence also considering cost-effectiveness [24, 25] remains a prerequisite for adoption by referring physicians, and PET-based endpoints in clinical trials will further support the generation of urgently needed evidence guiding PET implementation in clinical routine.

In meningioma, most participating sites reported the use of PET to select patients for radioligand therapies. Overall, such “theranostic” approaches based on a “see what you treat” strategy hold promise in the treatment of CNS tumours and particularly meningioma, while also here, robust evidence is missing to date [26, 27]. Radioligand therapy using [^177^Lu]Lu-DOTATATE is established in neuro-endocrine tumours based on pivotal clinical trial results [28, 29], and correlations between pre-treatment SSTR PET uptake and outcomes were observed [30]. Similar results were seen in a small retrospective case series of meningioma [31], although a clear relationship between pre-treatment SSTR expression, dosimetry, and treatment outcomes remains to be established, ideally by prospective clinical trials. In this regard, the LUMEN-1/EORTC-2334 (NCT06326190) trial aims to evaluate the efficacy of [^177^Lu]Lu-DOTATATE in patients with refractory meningioma after previous local therapy and is embedded in a comprehensive translational research program. Besides, the six largest centres (by number of SSTR PET per year) in the present survey reported radiotherapy planning as the most frequent indication, following data derived from small prospective trials showing an added layer of information when incorporating SSTR PET in radiotherapy planning for complex meningioma [32, 33].

While providing valuable insights in use of PET in neuro-oncology, the present survey has several limitations. The design is inherently linked to recall bias, missing responses, potential misinterpretation of questions, and replies potentially mirroring rather ideal practice than real-life scenarios. Specifically, there is a strong selection bias given that all invited sites are part of the EORTC-BTG network, which mainly consists of large tertiary care centres participating in clinical trials and with underrepresentation of sites in certain geographical areas such as Eastern Europe. Moreover, with only one third of invited sites replying, there was notable underreporting. Likely, participating sites were those using PET in clinical routine, as sites lacking such facilities might have been reluctant to answer negatively or in case of missing information on technical details outside their expertise. Both the pronounced selection as well as non-response biases might explain the high fraction of sites with high case numbers and those equipped with advanced technology such as combined PET-MRI. While the replies might reflect PET use in highly specialized settings at research-active sites, they underscore that PET-based endpoints are feasible in multicentric clinical brain tumour trials in the EORTC-BTG network. Nevertheless, comprehensive surveys also targeting non-academic centres are needed to define the application of PET in real-life settings. Furthermore, the medical specialties of respondents were heterogeneous, influencing the perceived patterns of PET use as reported in the survey, particularly when considering differing points of view between nuclear medicine physicians and referring specialties. Indeed, no attempts to independently validate replies by local nuclear medicine physicians or external insurance data were made. While participation by the local nuclear medicine physician was encouraged, they were only contacted directly if they were EORTC members affiliated with an EORTC-BTG site. Finally, the numbers in certain subgroups were small, precluding further inferential statistical analysis considering countries and subregions of participating sites.

In conclusion, the results of this survey show that implementation of PET is highly variable across institutions, entities, and indications throughout Europe. It has delivered valuable insights on factors hampering PET use in clinical routine with main takeaways including limited availability of tracers, high costs as well as a perceived redundancy of PET by referring physicians. To facilitate wider use and acceptance, besides increasing availability of tracers, more high-level evidence studies are needed, showing added value and ideally improved outcomes as well as cost-effectiveness in the management of patients with CNS tumours. The provided data in the present study show that PET-based endpoints are feasible in European multicentric neuro-oncological trials and provide a rational basis for the development of PET-based clinical trial designs. Given the potentially strong selection and non-response biases, future analyses involving also non-academic centres on national and international level are needed to ensure a more representative assessment of PET utilization in clinical routine.

## Supplementary Information

Below is the link to the electronic supplementary material.

Supplementary file1 Percentages of [18F]FDG PET examinations per (a) entity (b) indication at sites as estimated by survey participants. Abbreviations: CNS = Central Nervous System; PET = positron emission tomography; preOP/postOP = pre-/postoperative; RT = radiotherapy. (PDF 106 KB)

Supplementary file2 Survey items including question text, question type and options. (XLSX 16.1 KB)

Supplementary file3 Used amino acid PET tracers at participating sites. Abbreviations: [11C]MET = [11C]-methyl-L-methionine; [18F]FDOPA = 3,4-dihydroxy-6-[18F]-fluoro-L-phenylalanine; [18F]FET = O-(2-[18F]-fluoroethyl)-L-tyrosine; PET = positron emission tomography (PDF 183 KB)

## Data Availability

Underlying data can be provided upon reasonable request to the corresponding author and approval from relevant regulatory authorities.

## References

[1] Weller M, van den Bent M, Preusser M, Le Rhun E, Tonn JC, Minniti G, et al. EANO guidelines on the diagnosis and treatment of diffuse gliomas of adulthood. Nat Rev Clin Oncol. 2021;18:170–86. 33293629 10.1038/s41571-020-00447-z PMC7904519

[2] Goldbrunner R, Stavrinou P, Jenkinson MD, Sahm F, Mawrin C, Weber DC, et al. EANO guideline on the diagnosis and management of meningiomas. Neuro-Oncol. 2021;23:1821–34. 34181733 10.1093/neuonc/noab150 PMC8563316

[3] Le Rhun E, Guckenberger M, Smits M, Dummer R, Bachelot T, Sahm F, et al. EANO–ESMO clinical practice guidelines for diagnosis, treatment and follow-up of patients with brain metastasis from solid tumours. Ann Oncol. 2021;32:1332–47. 34364998 10.1016/j.annonc.2021.07.016

[4] Boellaard R, Delgado-Bolton R, Oyen WJG, Giammarile F, Tatsch K, Eschner W, et al. FDG PET/CT: EANM procedure guidelines for tumour imaging: version 2.0. Eur J Nucl Med Mol Imaging. 2015;42:328–54. 25452219 10.1007/s00259-014-2961-x PMC4315529

[5] Law I, Albert NL, Arbizu J, Boellaard R, Drzezga A, Galldiks N, et al. Joint EANM/EANO/RANO practice guidelines/snmmi procedure standards for imaging of gliomas using PET with radiolabelled amino acids and [18F]FDG: version 1.0. Eur J Nucl Med Mol Imaging. 2019;46:540–57. 30519867 10.1007/s00259-018-4207-9 PMC6351513

[6] Verger A, Tolboom N, Cicone F, Chang SM, Furtner J, Galldiks N, et al. Joint EANM/EANO/RANO/SNMMI practice guideline/procedure standard for PET imaging of brain metastases: version 1.0. Eur J Nucl Med Mol Imaging. 2025;52:1822–39. 10.1007/s00259-024-07038-5 PMC11928372 39762634

[7] Albert NL, Preusser M, Traub-Weidinger T, Tolboom N, Law I, Palmer JD, et al. Joint EANM/EANO/RANO/SNMMI practice guideline/procedure standards for diagnostics and therapy (theranostics) of meningiomas using radiolabeled somatostatin receptor ligands: version 1.0. Eur J Nucl Med Mol Imaging. 2024;51:3662–79. 10.1007/s00259-024-06783-x PMC11445317 38898354

[8] Galldiks N, Lohmann P, Aboian M, Barajas RF, Breen WG, Ivanidze J, et al. Update to the RANO working group and EANO recommendations for the clinical use of PET imaging in gliomas. Lancet Oncol. 2025. [in press] 10.1016/S1470-2045(25)00193-7 40744043

[9] Albert NL, Galldiks N, Ellingson BM, van den Bent MJ, Chang SM, Cicone F, et al. PET-based response assessment criteria for diffuse gliomas (PET RANO 1.0): a report of the RANO group. Lancet Oncol. 2024;25:e29–41. 38181810 10.1016/S1470-2045(23)00525-9 PMC11787868

[10] Albert NL, Galldiks N, Ellingson BM, van den Bent MJ, Chang SM, Cicone F, et al. Response assessment of brain metastases based on amino acid PET imaging: PET RANO BM 1.0 criteria. Nat Med. 2025;31:1424–30. 10.1038/s41591-025-03633-7 40341837

[11] Publications Office of the European Union. EuroVoc: Multilingual Thesaurus of the European Union, version 4.21. [Internet]. 2025. Available from: https://eur-lex.europa.eu/browse/eurovoc.html

[12] Parent EE, Johnson DR, Gleason T, Villanueva-Meyer JE. Neuro-Oncology practice clinical debate: FDG PET to differentiate glioblastoma recurrence from treatment-related changes. Neuro-Oncol Pract. 2021;8:518–25. 10.1093/nop/npab027 PMC8475205 34594566

[13] Telix Pharmaceuticals Ltd. Telix Provides Regulatory Update on TLX101-CDx. 2025 Apr 28 [cited 2025 May 1]; Available from: https://ir.telixpharma.com/static-files/e40a4bf3-7398-4dbd-82eb-feb10e9295fc

[14] Pafundi DH, Laack NN, Youland RS, Parney IF, Lowe VJ, Giannini C, et al. Biopsy validation of 18F-DOPA PET and biodistribution in gliomas for neurosurgical planning and radiotherapy target delineation: results of a prospective pilot study. Neuro-Oncol. 2013;15:1058–67. 23460322 10.1093/neuonc/not002 PMC3714146

[15] Harat M, Rakowska J, Harat M, Szylberg T, Furtak J, Miechowicz I, et al. Combining amino acid PET and MRI imaging increases accuracy to define malignant areas in adult glioma. Nat Commun. 2023;14:4572. 37516762 10.1038/s41467-023-39731-8 PMC10387066

[16] Gempt J, Bette S, Buchmann N, Ryang Y-M, Förschler A, Pyka T, et al. Volumetric analysis of F-18-FET-PET imaging for brain metastases. World Neurosurg. 2015;84:1790–7. 26255241 10.1016/j.wneu.2015.07.067

[17] Rachinger W, Stoecklein VM, Terpolilli NA, Haug AR, Ertl L, Pöschl J, et al. Increased 68Ga-DOTATATE uptake in PET imaging discriminates meningioma and tumor-free tissue. J Nucl Med. 2015;56:347–53. 25635133 10.2967/jnumed.114.149120

[18] Breen WG, Youland RS, Giri S, Jacobson SB, Pafundi DH, Brown PD, et al. Initial results of a phase II trial of 18F-DOPA PET-guided re-irradiation for recurrent high-grade glioma. J Neurooncol. 2022;158:323–30. 35583721 10.1007/s11060-022-04011-w

[19] Laack NN, Pafundi D, Anderson SK, Kaufmann T, Lowe V, Hunt C, et al. Initial results of a phase 2 trial of 18F-DOPA PET-Guided Dose-Escalated radiation therapy for glioblastoma. Int J Radiat Oncol. 2021;110:1383–95. 10.1016/j.ijrobp.2021.03.032 33771703

[20] Grosu A-L, Weber W, Graf E, Mix M, Wiehle R, Nestle U, et al. GLIAA: FET-PET- vs. MRI-based re-irradiation in recurrent glioblastoma. A prospective randomized trial. J Nucl Med. 2024;65:242119.

[21] Tsien CI, Pugh SL, Dicker AP, Raizer JJ, Matuszak MM, Lallana EC, et al. NRG Oncology/RTOG1205: A randomized phase II trial of concurrent bevacizumab and reirradiation versus bevacizumab alone as treatment for recurrent glioblastoma. J Clin Oncol Off J Am Soc Clin Oncol. 2023;41:1285–95. 10.1200/JCO.22.00164 PMC9940937 36260832

[22] Rahman R, Preusser M, Tsien C, Le Rhun E, Sulman EP, Wen PY, et al. Point/Counterpoint: the role of reirradiation in recurrent glioblastoma. Neuro-Oncol. 2025;27:7–12. 39527460 10.1093/neuonc/noae209 PMC11726241

[23] Preusser M, Kazda T, Le Rhun E, Sahm F, Smits M, Gempt J, et al. Lomustine with or without reirradiation for first progression of glioblastoma, LEGATO, EORTC-2227-BTG: study protocol for a randomized phase III study. Trials. 2024;25:366. 38849943 10.1186/s13063-024-08213-7 PMC11157762

[24] Rosen J, Werner J-M, Ceccon GS, Rosen EK, Wollring MM, Stetter I, et al. MRI and ^18^ F-FET PET for multimodal treatment monitoring in patients with brain metastases: A Cost-Effectiveness analysis. J Nucl Med. 2024;65:838–44. 38664020 10.2967/jnumed.123.266687

[25] Rosen J, Ceccon G, Bauer EK, Werner J-M, Tscherpel C, Dunkl V, et al. Cost effectiveness of 18F-FET PET for early treatment response assessment in glioma patients after adjuvant Temozolomide chemotherapy. J Nucl Med Off Publ Soc Nucl Med. 2022;63:1677–82. 10.2967/jnumed.122.263790 35422443

[26] Albert NL, Le Rhun E, Minniti G, Mair MJ, Galldiks N, Tolboom N, et al. Translating the theranostic concept to neuro-oncology: disrupting barriers. Lancet Oncol. 2024;25:e441–51. 39214115 10.1016/S1470-2045(24)00145-1

[27] Mair MJ, Tabouret E, Johnson DR, Sulman EP, Wen PY, Preusser M, et al. Radioligand therapies in meningioma: evidence and future directions. Neuro-Oncol. 2024;26:S215–28. 38702966 10.1093/neuonc/noae069 PMC11631075

[28] Strosberg J, El-Haddad G, Wolin E, Hendifar A, Yao J, Chasen B, et al. Phase 3 trial of ^177^ Lu-Dotatate for midgut neuroendocrine tumors. N Engl J Med. 2017;376:125–35. 28076709 10.1056/NEJMoa1607427 PMC5895095

[29] Strosberg JR, Caplin ME, Kunz PL, Ruszniewski PB, Bodei L, Hendifar A, et al. 177Lu-Dotatate plus long-acting octreotide versus high–dose long-acting octreotide in patients with midgut neuroendocrine tumours (NETTER-1): final overall survival and long-term safety results from an open-label, randomised, controlled, phase 3 trial. Lancet Oncol. 2021;22:1752–63. 34793718 10.1016/S1470-2045(21)00572-6

[30] Kratochwil C, Stefanova M, Mavriopoulou E, Holland-Letz T, Dimitrakopoulou-Strauss A, Afshar-Oromieh A, et al. SUV of [68Ga]DOTATOC-PET/CT predicts response probability of PRRT in neuroendocrine tumors. Mol Imaging Biol. 2015;17:313–8. 25319765 10.1007/s11307-014-0795-3

[31] Seystahl K, Stoecklein V, Schüller U, Rushing E, Nicolas G, Schäfer N, et al. Somatostatin receptor-targeted radionuclide therapy for progressive meningioma: benefit linked to 68Ga-DOTATATE/-TOC uptake. Neuro-Oncol. 2016;18:1538–47. 27106404 10.1093/neuonc/now060 PMC5063513

[32] Milker-Zabel S, Zabel-du Bois A, Henze M, Huber P, Schulz-Ertner D, Hoess A, et al. Improved target volume definition for fractionated stereotactic radiotherapy in patients with intracranial meningiomas by correlation of CT, MRI, and [68Ga]-DOTATOC-PET. Int J Radiat Oncol. 2006;65:222–7. 10.1016/j.ijrobp.2005.12.006 16488553

[33] Nyuyki F, Plotkin M, Graf R, Michel R, Steffen I, Denecke T, et al. Potential impact of 68Ga-DOTATOC PET/CT on stereotactic radiotherapy planning of meningiomas. Eur J Nucl Med Mol Imaging. 2010;37:310–8. 19763565 10.1007/s00259-009-1270-2

