# Supplementary figures and images for: Availability and use of PET in patients with brain tumours – a European Organisation for Research and Treatment of Cancer - Brain Tumour Group (EORTC-BTG) survey
Source: Eur J Nucl Med Mol Imaging. 2025 Jun 4;52(12):4627–38. doi: 10.1007/s00259-025-07366-0 (PMC12491378; doi:10.1007/s00259-025-07366-0)

Amino acid PET tracers (multiple answers possible, n = 59)

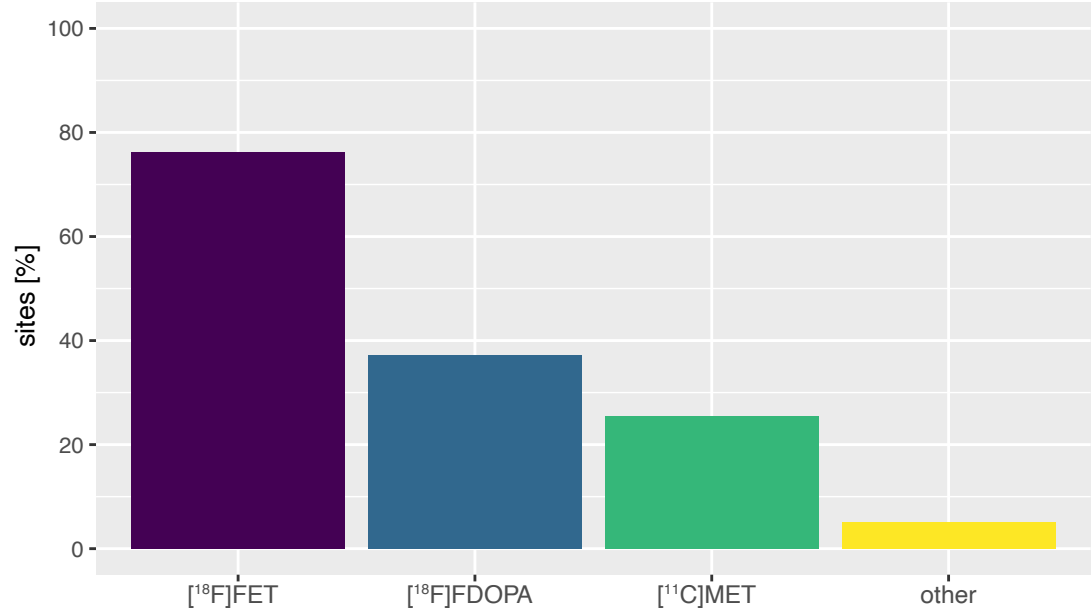

Supplement: Supplementary file 1 — Supplementary file1 Percentages of [18F]FDG PET examinations per (a) entity (b) indication at sites as estimated by survey participants. Abbreviations: CNS = Central Nervous System; PET = positron emission tomography; preOP/postOP = pre-/postoperative; RT = radiotherapy. (PDF 106 KB) [file 259_2025_7366_MOESM1_ESM.pdf]

**a** [<sup>18</sup>F]FDG PET by entity (n = 23)

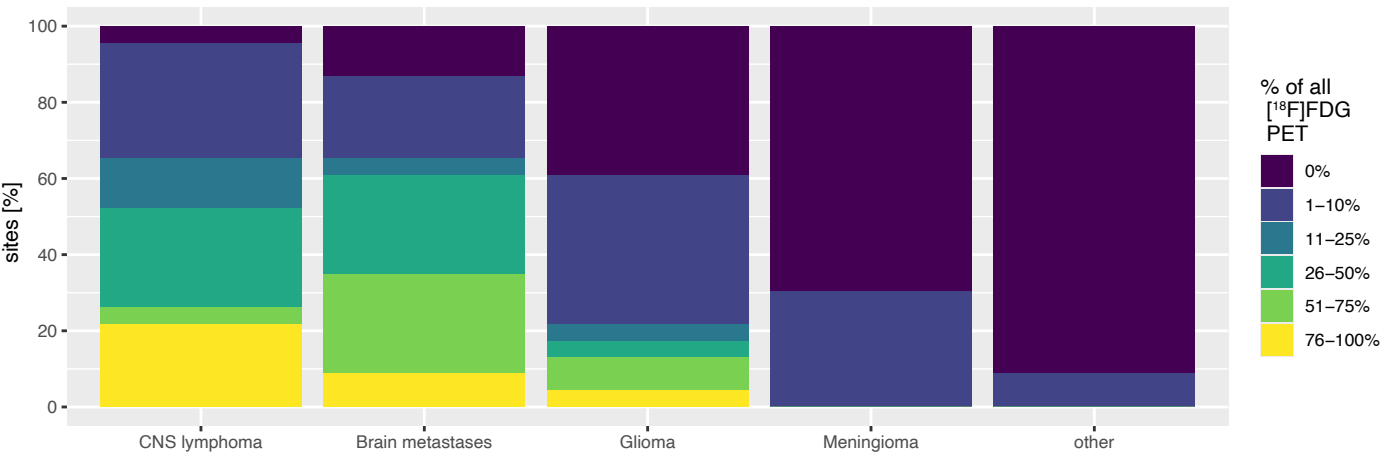

**b** [<sup>18</sup>F]FDG PET by indication (n = 23)

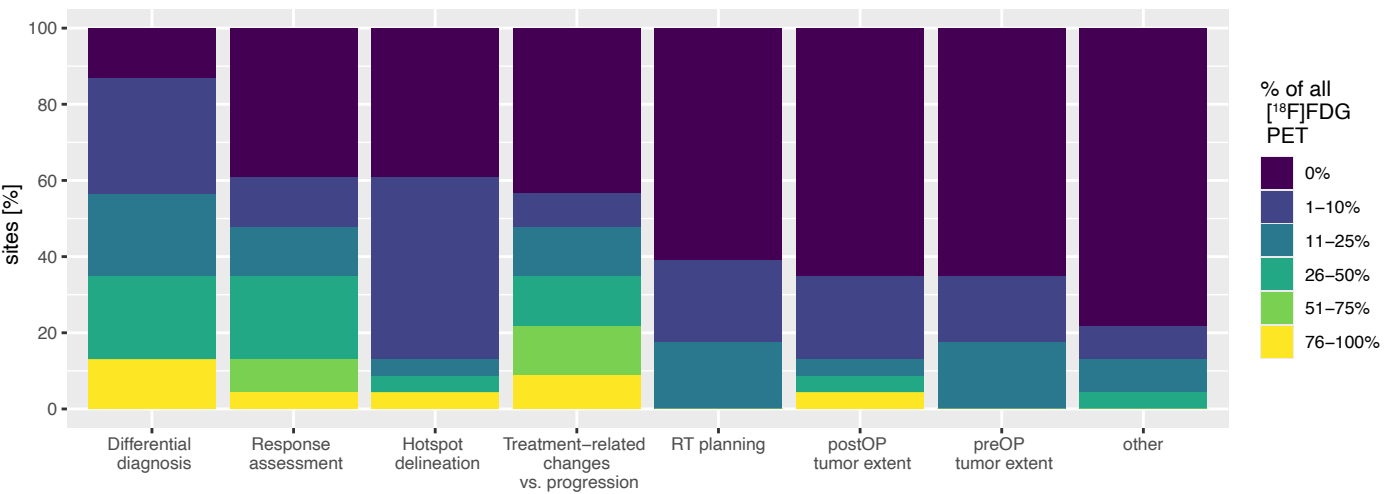

Supplement: Supplementary file 3 — Supplementary file3 Used amino acid PET tracers at participating sites. Abbreviations: [11C]MET = [11C]-methyl-L-methionine; [18F]FDOPA = 3,4-dihydroxy-6-[18F]-fluoro-L-phenylalanine; [18F]FET = O-(2-[18F]-fluoroethyl)-L-tyrosine; PET = positron emission tomography (PDF 183 KB) [file 259_2025_7366_MOESM3_ESM.pdf]
